# Multi-Sensor Heterogeneous Signal Fusion Transformer for Tool Wear Prediction

**DOI:** 10.3390/s25154847

**Published:** 2025-08-06

**Authors:** Ju Zhou, Xinyu Liu, Qianghua Liao, Tao Wang, Lin Wang, Pin Yang

**Affiliations:** 1 Tech X Academy, Shenzhen Polytechnic University, Shenzhen 518055, China; zhouju@szpu.edu.cn (J.Z.); liaoqianghua@szpu.edu.cn (Q.L.); 2 Shenzhen Institute of Advanced Technology, Chinese Academy of Sciences, Shenzhen 518055, China; lin.wang1@siat.ac.cn; 3 College of Electronic and Information Engineering, Southwest University, Chongqing 400715, China; liu1223xy@email.swu.edu.cn; 4 School of Mechanical Engineering and Automation, University of Science and Technology Liaoning, Anshan 114044, China; yangpin1030@163.com

**Keywords:** tool wear prediction, multi-sensor signal, feature fusion, Transformer models, deep learning

## Abstract

In tool wear monitoring, the efficient fusion of multi-source sensor signals poses significant challenges due to their inherent heterogeneous characteristics. In this paper, we propose a Multi-Sensor Multi-Domain feature fusion Transformer (MSMDT) model that achieves precise tool wear prediction through innovative feature engineering and cross-modal self-attention mechanisms. Specifically, we first develop a physics-aware feature extraction framework, where time-domain statistical features, frequency-domain energy features, and wavelet packet time–frequency features are systematically extracted for each sensor type. This approach constructs a unified feature matrix that effectively integrates the complementary characteristics of heterogeneous signals while preserving discriminative tool wear signatures. Then, a position-embedding-free Transformer architecture is constructed, which enables adaptive cross-domain feature fusion through joint global context modeling and local feature interaction analysis to predict tool wear values. Experimental results on the PHM2010 demonstrate the superior performance of MSMDT, outperforming state-of-the-art methods in prediction accuracy.

## 1. Introduction

In Computer Numerical Control (CNC) machining systems, cutting tools are critical components. Accurate tool wear prediction is important for two reasons: First, inadequate analysis of sensor signals (including acoustic emission, force, and vibration) leads to premature tool replacement, resulting in underutilization of remaining tool life. Second, undetected latent wear can degrade product quality and may even cause tool breakage and machine stoppage in severe cases [1,2,3]. Consequently, accurate tool wear prediction enables optimal resource utilization, reduces production costs, and prevents machining incidents.

Current monitoring approaches primarily rely on two types of signals to predict tool wear conditions: direct measurement signals (e.g., visual images [4] and laser contours [5]) and indirect inference signals (e.g., cutting force [6], vibration [7], acoustic emission [8], and spindle motor load [9]). However, monitoring systems based on single-sensor signals exhibit inherent limitations: direct signals remain vulnerable to environmental interference, while indirect signals, being uni-dimensional in nature, cannot fully characterize complex wear mechanisms. The fundamental drawback of single-sensor approaches lies in their ability to capture only localized wear features, rendering them incapable of overcoming environmental sensitivity and establishing robust correlations with wear states. These limitations consequently compromise the reliability and generalizability of monitoring systems.

To overcome the bottlenecks of single-sensor systems, the use of multi-sensors to acquire diverse signals for monitoring tool wear conditions has emerged as a mainstream research trend. By integrating signals such as force, vibration, and acoustic emission, researchers are able to extract more comprehensive information about the tool wear process [10,11]. For example, Silva et al. [12] demonstrated the feasibility of fusing acoustic emission and power signals for tool wear monitoring, revealing that the multi-sensor approach yields significantly superior results compared to single-sensor methods. However, the data acquired from different sensor sources inherently exhibit heterogeneity in terms of sampling rates, physical units, and information granularity. The key challenge lies in developing effective fusion strategies that can fully exploit the complementary information while addressing the dimensional inconsistencies among these heterogeneous data streams.

With the rapid development of computer technology, numerous machine learning and deep learning approaches have been introduced into the field of tool wear monitoring [13]. These studies primarily consist of two steps: firstly, feature engineering, which involves extracting key features from signals, and subsequently, training high-performance prediction models based on these features.

In feature engineering research for tool wear monitoring, researchers have employed various signal processing methods to extract features from sensor data. For vibration signals, Rmili et al. [14] extract average power as a feature to represent tool wear state, while Elangovan et al. [15] focus on statistical feature extraction, and Huang et al. [7] utilize the Short-Time Fourier Transform (STFT) for time–frequency analysis to obtain key features. For vibration and acoustic signals, Lin et al. [16] apply the Fast Fourier Transform (FFT) for feature extraction, while Rafezi et al. [17] not only extract time-domain statistical features but also analyze spectral characteristics and employ wavelet packet decomposition to explore specific frequency band information. However, these feature extraction methods exhibit certain limitations. First, the extracted feature types remain relatively simplistic, with different methods being applied to different signals, lacking comprehensive exploration of the multi-dimensional characteristics. Second, the feature design relies on manual expertise, resulting in limited effectiveness and adaptability when facing different signal types or varying working conditions. More critically, these methods struggle to automatically capture deep correlation features between signals, leading to insufficient information utilization. This feature extraction homogeneity constrains the generalization capability of monitoring systems in complex industrial scenarios.

In tool wear modeling research, researchers have progressively shifted from traditional machine learning methods to deep learning models. The deep Convolutional Neural Network (CNN) model developed by Fu et al. [18] demonstrates superior performance to conventional Support Vector Machine (SVM) in drilling process monitoring. By incorporating the deep Residual Network (ResNet) and Long Short-Term Memory (LSTM) network, Sun et al. [19] propose the hybrid ResNet-LSTM architecture, which achieves more accurate multi-step prediction through multi-sensor signal inputs. Wang et al. [20] construct the CNN-BiLSTM model, which further enhances monitoring performance. Although these deep learning approaches have made significant advancements, current models exhibit notable limitations: CNN-based methods are constrained by local receptive fields, hindering their ability to model global dependencies in long sequences; LSTM and its variants, while proficient in processing temporal data, suffer from inherently inefficient training due to recursive computation. Furthermore, these conventional network architectures lack sufficient capability for differentiated feature processing of multi-source heterogeneous sensor signals, failing to adaptively capture inter-feature relationships and achieve effective fusion.

To tackle the aforementioned issues, we propose a Multi-Sensor Multi-Domain feature fusion Transformer (MSMDT) approach for tool wear prediction. For heterogeneous signals from multi-sensors, including acoustic emission, cutting force, and vibration, this approach first introduces a unified physical perception-based feature extraction method. This method systematically processes each sensor’s signal through complementary time-domain statistical analysis, frequency-domain energy representation, and wavelet packet time–frequency decomposition. These multi-domain features are then integrated into a compact and comprehensive feature matrix, which not only preserves the physical interpretability of specific sensors but also enables cross-modal correlation analysis. Furthermore, to comprehensively model the complex nonlinear interactions within the feature matrix, we develop the MSMDT framework based on the Transformer architecture. By designing a feature fusion encoder and eliminating the position embedding process, the MSMDT enables efficient parallel processing and integration of multi-sensor multi-domain features. This facilitates information interaction among these features and ultimately enhances the accuracy of tool wear prediction. The main contributions of this paper are as follows:

1. Multi-domain feature fusion strategy for multi-sensor heterogeneous signals: Effectively integrates multi-source industrial sensor signals through collaborative modeling of time-domain, frequency-domain, and time–frequency-domain features, providing feature-re-engineering-free extensibility to diverse sensor types while leveraging complementary signal characteristics.
2. Position-embedding-free MSMDT network design: Enables parallel processing and real-time collaborative prediction of cross-sensor information, enhancing effective feature fusion for heterogeneous temporal signals.
3. Breakthrough in single-sensor and single-feature dependency limitations: The proposed method can adaptively extract deep-level features that characterize tool wear and automatically predict its progression, achieving promising results.

The rest of this paper is structured as follows. Section 2 presents the related work, Section 3 describes the proposed MSMDT, Section 4 presents the experimental results and comparative analysis, and Section 5 summarizes and discusses this study.

## 2. Related Work

In tool wear monitoring research, cutting force, vibration, and acoustic emission signals reflect wear states from distinct perspectives. The cutting force signals characterize mechanical load variations at the tool–workpiece interface, while vibration and acoustic emission signals capture structural dynamics and microscopic material damage, respectively. Current studies employ different feature extraction strategies tailored to each signal type: time-domain statistical features such as root mean square and peak-to-peak values are primarily extracted from force signals to quantify overall cutting force intensity [21,22]; frequency-domain features are applied to vibration signals to track energy shifts in specific frequency bands [23,24,25]; and time–frequency features obtained through wavelet packet transform analyze the non-stationary characteristics of acoustic emission signals [26,27].

Although these domain-specific methods demonstrate effectiveness in individual signal processing, it does not imply other signal domains lack valuable information. For instance, cutting force signals contain meaningful frequency components reflecting tool resonance, and time-domain kurtosis of vibration effectively detects impact events during wear initiation. Therefore, single-domain features may fail to comprehensively characterize complex wear progression, and cross-sensor feature correlations remain underutilized. This limitation necessitates a unified multi-domain feature extraction framework capable of simultaneous time-domain, frequency-domain, and time–frequency-domain characterization of multi-sensor signals. Such an integrated approach preserves the physical significance of each signal modality while exploiting feature complementarity to achieve more complete wear state representation, thereby providing enhanced information infrastructure for subsequent feature fusion and prediction modeling.

Among various networks, LSTM networks effectively address the vanishing gradient problem of traditional recurrent neural networks (RNNs) through gating mechanisms and memory cell design, making them suitable for capturing long-term temporal dependencies in tool wear processes [28,29,30]. To better use both forward and backward time-series information, bidirectional LSTM (BiLSTM) has been introduced to capture the dynamic evolution of tool wear more comprehensively and improve the accuracy [31,32]. Currently, hybrid models combining LSTM with other network architectures have become a research focus, such as integrating CNNs for simultaneous local feature extraction and temporal dependency modeling or incorporating attention mechanisms to enhance key feature selection, with these hybrid approaches demonstrating superior robustness and performance in wear prediction [20,33,34].

Although LSTM performs well in tool wear prediction, its inherent sequence dependency leads to low computational efficiency and difficulty in establishing global dependencies across sensors. In contrast, the Transformer architecture demonstrates advantages in multi-sensor data processing [35]. Its core multi-head attention mechanism can process all sequential data in parallel, significantly enhancing computational efficiency. Meanwhile, through the self-attention mechanism, it dynamically models feature correlations at arbitrary positions, making it suitable for capturing complex interactions among multi-source sensor signals. Recently, the Transformer architecture has achieved notable success in the field of fault diagnosis [36,37,38].

Therefore, this study proposes the MSMDT to predict tool wear states, which addresses the limitations of current research from two aspects. On the one hand, by constructing a multi-domain feature extraction strategy, it effectively tackles the issues of heterogeneous multi-sensor signals and inadequate information utilization. For different temporal signals, the same feature extraction strategy is employed to extract meaningful information, which is then integrated into a compact feature matrix. This approach not only ensures full utilization of information but also achieves a multi-modal consistent representation of tool wear states, providing a unified input format for the model. On the other hand, the MSMDT utilizes the Transformer architecture to effectively fuse multi-domain features from multi-sensors, establishing a reliable wear prediction model. Leveraging the Transformer, long-range dependencies of tool wear states can be established without recursion, and the importance of features extracted from different sensor signals is automatically assigned through the self-attention mechanism, thereby adaptively extracting features highly relevant to the tool wear state. The unified feature extraction strategy and extensible prediction model of MSMDT offer a promising solution for future scenarios involving different sensors and operating conditions.

## 3. MSMDT

**Figure 1:**
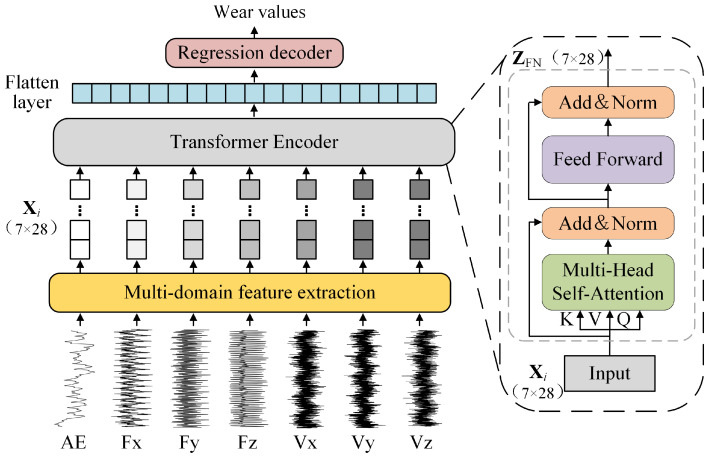
Structure of MSMDT.

The structure of the proposed MSMDT model is shown in Figure 1. The MSMDT framework primarily consists of four key processes: multi-sensor signal input, multi-domain feature extraction, Transformer encoder processing, and regression decoder output.

To overcome the limitation of poor anti-interference ability in single-sensor systems, a multi-sensor signal input structure is designed. Here, AE represents the acoustic emission (AE) signal, while $F_{x}$, $F_{y}$, and $F_{z}$ denote the cutting force signals collected in the three orthogonal directions (X, Y, Z), respectively. Similarly, $V_{x}$, $V_{y}$, and $V_{z}$ correspond to the vibration signals in the same directions. These seven-channel signals are collectively input into the MSMDT model. In the multi-domain feature extraction phase, 28 features are extracted from each signal to reduce data dimensionality while preserving critical tool wear-related information, forming a high-density feature matrix $X_{i}\in R^{7\;\times \;28}$. These features cover multiple domains, including the time domain, frequency domain, and wavelet packet decomposition time–frequency domain. Unlike traditional tool prediction models, our approach utilizes a Transformer-based backbone network to process the feature matrix $X_{i}$. This is achieved through the encoder module of the Transformer, the detailed structure of which is illustrated on the right side of Figure 1. Notably, the MSMDT model omits position embeddings compared to standard Transformer architectures to decouple multi-sensor features and enhance system performance. After extracting deep features in the Transformer encoder, a flattening layer transforms these features into a 196 × 1 vector to adapt to the MSMDT-specific regression decoder. As tool wear prediction is a regression task, the decoder comprises three fully connected layers with node configurations of 196→128, 128→64, and 64→1, ultimately outputting the predicted tool wear value.

### 3.1. Multi-Sensor Signal Input

During the machining process, relying solely on a single sensor may lead to unreliable tool wear assessment due to inherent sensor limitations, signal transmission issues, and external noise interference. To overcome these limitations, multi-sensor fusion technology has become the predominant approach for comprehensive tool condition monitoring. This study focuses on three complementary sensor modalities: acoustic emission sensor, cutting force sensor, and vibration sensor.

- Acoustic emission sensors are cost-effective and readily available, but they are prone to mechanical noise and require complex signal analysis.
- Cutting force sensors are highly sensitive to tool wear signals and provide accurate monitoring, albeit at a higher cost.
- Vibration sensors offer easy installation, low cost, and abundant information, but they are susceptible to environmental interference.

Currently, these three sensor types are widely adopted in CNC machining systems for tool wear monitoring due to their complementary sensing capabilities. Among them, the acoustic emission signal contains one-dimensional data, and the force and vibration signals contain three directional components. This means that the three sensors have a total of seven signal channels. However, the heterogeneous nature of these signals poses significant challenges for integrated analysis: these signals have different units of measurement and ranges of numerical variation; they contain sensor-specific noise patterns and interference characteristics; and they respond to tool wear with varying sensitivity and latency. This heterogeneity makes it difficult to establish consistent correlations between the raw signals and tool wear states. To address these challenges, a unified multi-domain feature extraction framework is proposed to process diverse temporal signals from multi-source industrial sensors while preserving their essential wear-related information. This framework adaptively handles heterogeneous signals and extracts discriminative features for accurate tool condition assessment.

### 3.2. Multi-Domain Feature Extraction

Before feature extraction, the raw signals undergo two preprocessing steps: outlier removal via the quartile method and elimination of direct current (DC) components. The outlier removal ensures data integrity by excluding abnormal measurements, while DC component elimination prepares signals for effective frequency-domain feature extraction. These operations enhance signal quality for subsequent multi-domain feature analysis under varying operational conditions, providing optimized inputs for the Transformer model.

To overcome the limitation of redesigning feature engineering processes when signals or their combinations change, this study develops a unified multi-domain feature extraction framework. This framework further fuses features into a common representation, maximally extracting discriminative features from diverse signals across multiple dimensions for effective tool wear state prediction. The multi-domain feature extraction comprises three distinct domains (time domain, frequency domain, and time–frequency domain), totaling 28 features, with detailed mathematical formulations and physical explanations provided in Table 1. Specifically, the following are considered:

- Time domain (9 features): Statistical descriptors of signal amplitudes, including standard deviation, variance, peak-to-peak, RMS value, skewness coefficient, kurtosis coefficient, crest factor, margin factor, and waveform factor.
- Frequency domain (3 features): Spectral characteristics including center gravity frequency, frequency variance, and mean square frequency.
- Time–frequency domain (16 features): Wavelet packet decomposition energies across 16 sub-bands.

For seven input signals, the use of a multi-domain feature extraction approach yields a compact and informative feature matrix (7 × 28) that combines physical interpretability with data-driven robustness. Wavelet-derived features compensate for the limitations of a single time- or frequency-domain analysis, and the low dimensionality of the matrix ensures computational efficiency while retaining the ability to discriminate tool wear. More importantly, the inherent adaptability of the framework allows for the free integration of additional sensor inputs or machining conditions through its standardized feature extraction approach. These high-quality fused features are subsequently fed into the Transformer encoder module, where their inherent correlations and global dependencies are further extracted through self-attention mechanisms to establish a more comprehensive wear state representation.

**Table 1:** Multi-domain feature extraction methods.

Domain	Feature	Expression	Explanation
Time domain (9 features)	Standard deviation	$x_{std}=\sqrt{\frac{\sum_{i=1}^{n}{\left(x_{i}-\bar{x}\right)}^{2}}{n-1}}$	Sample standard deviation
	Variance	$x_{var}=\frac{\sum_{i=1}^{n}{\left(x_{i}-\bar{x}\right)}^{2}}{n-1}$	Sample variance
	Peak-to-peak	$x_{p-p}=x_{max}-x_{min}$	Difference between extrema
	RMS value	$x_{rms}=\sqrt{\frac{1}{n}\sum_{i=1}^{n}x_{i}^{2}}$	Root mean square
	Skewness coefficient	$x_{\gamma }=\frac{1}{n}\frac{\sum_{i=1}^{n}{\left(x_{i}-\bar{x}\right)}^{3}}{x_{std}^{3}}$	3rd standardized moment
	Kurtosis coefficient	$x_{g}=\frac{1}{n}\frac{\sum_{i=1}^{n}{\left(x_{i}-\bar{x}\right)}^{4}}{x_{std}^{4}}-3$	Excess kurtosis
	Crest factor	$x_{c}=\frac{|x_{p-p}|}{x_{rms}}$	Peak-to-RMS ratio
	Margin factor	$x_{m}=\frac{x_{p-p}}{\frac{1}{n}\sum_{i=1}^{n}x_{i}}$	Peak-to-average ratio
	Waveform factor	$x_{w}=\frac{x_{rms}}{\frac{1}{n}\sum_{i=1}^{n}x_{i}}$	RMS-to-average ratio
Frequency domain (3 features)	Center gravity frequency	$f_{cg}=\frac{\sum_{i=1}^{n}f_{i}p_{i}}{\sum_{i=1}^{n}p_{i}}$	Spectral centroid
	Frequency variance	$f_{v}=\frac{\sum_{i=1}^{n}{\left(f_{i}-f_{cg}\right)}^{2}p_{i}}{\sum_{i=1}^{n}p_{i}}$	Spectral spread
	Mean square frequency	$f_{ms}=\frac{\sum_{i=1}^{n}f_{i}^{2}p_{i}}{\sum_{i=1}^{n}p_{i}}$	Spectral RMS
Time–frequency domain (16 features)	Wavelet packet energy	$E_{wp}=\sum_{i=1}^{n}{\left|w_{j,k}\left[i\right]\right|}^{2}$	Decomposition energy

Common symbols: $x_{i}$—data point; $\bar{x}$—sample mean; *n*—data count; $f_{i}$—frequency component; $p_{i}$—power spectral density; $w_{j,k}\left[i\right]$—wavelet coefficient at level *j*, node *k*.

### 3.3. Backbone Structure

#### 3.3.1. Input Representation and Tokenization

The encoder input is a multi-domain feature matrix $X_{i}\in R^{7\;\times \;28}$, where each sensor channel’s feature vector is mapped to an independent token. Specifically, for the *k*-th sensor’s feature vector,

(1) $${\mathrm{Token}}_{k}=X_{i}\left[k,:\right]\in R^{28},\;k=1,2,\ldots ,7$$

The input is consequently represented as a token sequence:

(2) $$X_{\mathrm{tokens}}=\left[{\mathrm{Token}}_{1};{\mathrm{Token}}_{2};\ldots ;{\mathrm{Token}}_{7}\right]\in R^{7\;\times \;28}$$

This representation enables parallel processing of multi-sensor features, where each token encapsulates time-domain statistical features, frequency-domain energy spectrum characteristics, and wavelet packet energy features, physically representing the parallel characterization of multi-sensor features.

#### 3.3.2. Position-Embedding-Free Inter-Sensor Interaction

Fundamentally distinct from the traditional Transformer architecture, this study removes the position embedding operation. The decision is theoretically grounded in three aspects: Firstly, in tool wear monitoring tasks, the physical arrangement order of sensors generally does not contain semantically meaningful information, and forcibly introducing positional indices may cause feature entanglement, thereby damaging feature independence. Secondly, the self-attention mechanism intrinsically possesses the capability to autonomously learn inter-sensor correlations without requiring predefined spatial dependencies. Thirdly, when integrated with the tokenization strategy described in Section 3.3.1, this architecture exhibits remarkable scalability, where changes in the number of sensors only necessitate adjustments to the token count while preserving consistent encoding rules, thereby obviating the need for recalibration of position embeddings. The effectiveness of this position embedding removal strategy is further validated through quantitative ablation studies in our experimental section (see Section 4.3.3).

#### 3.3.3. Encoder Computational Architecture

The encoder architecture implements hierarchical feature transformation through stacked self-attention and feed-forward layers, maintaining sensor-specific information flow while enabling cross-modal interaction.

(1) Multi-Head Self-Attention Mechanism

The core operator establishes latent sensor correlations via query–key–value projection:

(3) $$\begin{array}{cccc} Q & =X_{\mathrm{tokens}}\;W_{Q}, & W_{Q} & \in R^{28\;\times \;d_{k}}\\ K & =X_{\mathrm{tokens}}\;W_{K}, & W_{K} & \in R^{28\;\times \;d_{k}}\\ V & =X_{\mathrm{tokens}}\;W_{V}, & W_{V} & \in R^{28\;\times \;d_{k}} \end{array}$$

(4) $$\mathrm{Attention}\left(Q,K,V\right)=\mathrm{Softmax}\left(\frac{QK^{⊤}}{\sqrt{d_{k}}}\right)V\in R^{7\;\times \;d_{k}}$$

where $d_{k}$ controls the latent interaction space dimension. This design complements the position embedding removal in Section 3.3.2, enabling physics-guided feature learning.

(2) Layer-wise Processing

Each encoder layer *l* processes token sequences through two cascaded sublayers with residual connections and layer normalization:

(5) $$Z^{\left(l\right)}=\mathrm{LayerNorm}\left(X_{\mathrm{tokens}}^{\left(l\right)}+\mathrm{MHA}\left(X_{\mathrm{tokens}}^{\left(l\right)}\right)\right)\;$$

(6) $$X_{\mathrm{tokens}}^{\left(l+1\right)}=\mathrm{LayerNorm}\left(Z^{\left(l\right)}+F\left(Z^{\left(l\right)}\right)\right)\;$$

where MHA(·) denotes the multi-head attention mechanism defined in Equation (4) and LayerNorm(·) represents layer normalization, which standardizes each token’s features by subtracting the mean and dividing by the standard deviation.

The feed-forward network F can be represented as folows:

(7) $$F\left(x\right)=\mathrm{GELU}\left(xW_{1}+b_{1}\right)W_{2}+b_{2},\;\left\{\begin{array}{c} W_{1}\in R^{28\;\times \;112}\\ W_{2}\in R^{112\;\times \;28} \end{array}\right.$$

Here, the Gaussian Error Linear Unit (GELU) function GELU(x)=xΦ(x) serves as the activation function. The final output $X_{\mathrm{tokens}}^{\left(\mathrm{out}\right)}\in R^{7\;\times \;28}$ maintains thedimensional consistency.

### 3.4. Regression Decoder

The decoder module maps deep features from the Transformer encoder to scalar predictions of tool wear. The encoder output $X_{\mathrm{tokens}}^{\left(\mathrm{out}\right)}\in R^{7\;\times \;28}$ is first flattened into a 1D vector using

(8) $$z=\mathrm{Flatten}\left(X_{\mathrm{tokens}}^{\left(\mathrm{out}\right)}\right)\in R^{196}$$

The regression decoder then processes the features through three fully connected layers:

(9) $$h_{1}=\mathrm{ReLU}\left(W_{3}z+b_{3}\right)$$

(10) $$h_{2}=\mathrm{ReLU}\left(W_{4}h_{1}+b_{4}\right)$$

(11) $$\hat{y}=W_{5}h_{2}+b_{5}$$

In Equation (8), the flatten operation reshapes the 7 × 28 matrix into a 196-dimensional vector in row-major order. Equations (9)–(11) define the computational workflow of the fully connected layers, where $W_{3}\in R^{128\;\times \;196}$ and $b_{3}\in R^{128}$ represent the weight matrix and bias vector for the first layer, $W_{4}\in R^{64\;\times \;128}$ and $b_{4}\in R^{64}$ those for the second layer, and $W_{5}\in R^{1\;\times \;64}$ and $b_{5}\in R^{1}$ those for the output layer. The Rectified Linear Unit (ReLU) activation function ReLU(x)=max(0,x) provides nonlinear transformations between layers.

The model is trained using a combined loss function of L1 loss and Mean Squared Error (MSE). They are calculated as follows:

(12) $$L_{L1}=\frac{1}{N}\sum_{i=1}^{N}\left|y_{i}-{\hat{y}}_{i}\right|$$

(13) $$L_{\mathrm{MSE}}=\frac{1}{N}\sum_{i=1}^{N}{\left(y_{i}-{\hat{y}}_{i}\right)}^{2}$$

The composite loss function combines these two components:

(14) $$L_{\mathrm{total}}=\alpha L_{L1}+\left(1-\alpha \right)L_{\mathrm{MSE}}$$

In Equations (12) and (13), $y_{i}$ denotes the actual tool wear value of the *i*-th sample, ${\hat{y}}_{i}$ denotes the predicted value, and *N* represents the batch size. α∈[0,1] controls the relative contribution of L1 and MSE losses in Equation (14). Parameter updates are performed via backpropagation:

(15) $$\Theta^{\left(t+1\right)}=\Theta^{\left(t\right)}-\eta \nabla_{\Theta }L_{\mathrm{total}}$$

where $\Theta =\{W_{3},b_{3},W_{4},b_{4},W_{5},b_{5}\}$ represents the trainable parameter set, η is the learning rate, and *t* indexes the training iteration. This hierarchical dimensionality reduction architecture enables robust mapping from the high-dimensional feature space to tool wear measurements in physical units.

## 4. Experimentation and Validation

### 4.1. Experimental Setup

#### 4.1.1. Datasets and Preprocessing

To validate the effectiveness of the proposed MSMDT in addressing the fundamental challenges of sensor data fusion for tool wear prediction, all experiments in this study are conducted on the publicly available PHM2010 dataset [39]. This choice is particularly valuable for demonstrating the robustness of our approach, as benchmarking on standardized public data provides a rigorous and reproducible foundation to evaluate generalization capabilities across diverse sensor scenarios. In this dataset, each tool executes a 108 mm stroke in the X direction, involving a total of 315 cuts across six tools. The machining process employs the following cutting parameters: spindle speed of 10,400 rpm, feed rate of 1555 mm/min, Y-axis depth of cut of 0.125 mm, and Z-axis depth of cut of 0.2 mm. The signals are initially amplified and subsequently captured by a data acquisition card operating at a sampling frequency of 50 kHz. The sensor-acquired signals comprise seven-dimensional data, including acoustic emission, cutting forces (in the X, Y, and Z directions), and vibrations (in the X, Y, and Z directions), with corresponding tool wear values recorded simultaneously. In this study, we use three available tools (C1, C4, and C6) from the public PHM2010 dataset [39] for analysis and validation.

**Figure 2:**
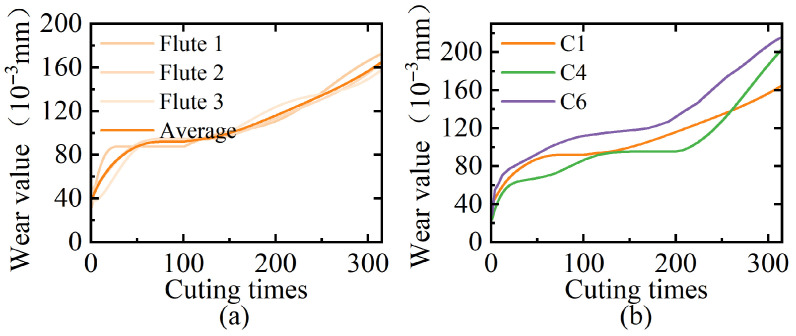
(**a**) Three flank wear measurements of C1; (**b**) wear progression curves for C1, C4, and C6.

The quantification of tool wear follows a standardized protocol in which a wear label for each cycle is generated by averaging three flank (three-flute ball end mill) wear measurements. Using C1 as an example, Figure 2a shows the wear curves on the three flanks as well as the final wear pattern. Figure 2b shows the wear progression curves for tool groups C1, C2, and C3, demonstrating a characteristic three-phase wear pattern across all cases: (1) Initial wear phase: Characterized by a sharp slope increase in wear values. (2) Steady wear phase: Marked by stable tool wear values with smaller gradient amplitudes. (3) Severe wear phase: Exhibiting an abrupt slope surge until functional failure. All curves maintain strict monotonic non-decreasing behavior throughout the cutting process.

The preprocessing workflow comprises three critical stages: **label construction** computes cycle labels through averaging flank wear measurements; **feature engineering** extracts time-domain, frequency-domain, and time–frequency-domain features following Table 1 to build a feature matrix; and **cross-validation** adopts a hold-out strategy, as shown in Table 2, where two tool groups (630 samples) train the model and the remaining group (315 samples) independently tests model generalizability.

#### 4.1.2. Model Configuration and Hyperparameters

The experimental platform utilizes an Intel I7-13650H processor and an NVIDIA RTX 4060 GPU, with the model implemented using the PyTorch framework (Version: 2.3.1). For model configuration, we utilize the Adam optimizer with a learning rate of 0.001. The batch size is set to 32 to optimally balance GPU memory constraints and training efficiency. Through empirical analysis of the loss curve, we confirm that 500 training epochs ensure model convergence. A dropout rate of 0.1 is applied to prevent overfitting in the MSMDT architecture while maintaining critical features. The loss function weight α is 0.5, which is determined through grid search experiments in the range of 0.1 to 0.9, reflecting equal importance between L1 and MSE loss components. To guarantee reproducibility, we fix the random seed at 3407. All experimental results in this study can be reproduced using these key parameters.

#### 4.1.3. Evaluation Metrics

Mean Absolute Error (MAE) and Root Mean Square Error (RMSE) are adopted, and they are calculated as follows:

(16) $$\mathrm{MAE}=\frac{1}{M}\sum_{i=1}^{M}\left|y_{i}-{\hat{y}}_{i}\right|$$

(17) $$\mathrm{RMSE}=\sqrt{\frac{1}{M}\sum_{i=1}^{M}{\left(y_{i}-{\hat{y}}_{i}\right)}^{2}}$$

where $y_{i}$ denotes the ground truth wear value, ${\hat{y}}_{i}$ represents the predicted value, and *M* indicates the number of test samples. Lower metric values correspond to higher prediction accuracy. In addition, the absolute error between the predicted and actual values is calculated and visualized to show the model prediction performance.

### 4.2. Visualization and Analysis of Multi-Sensor Features

**Figure 3:**
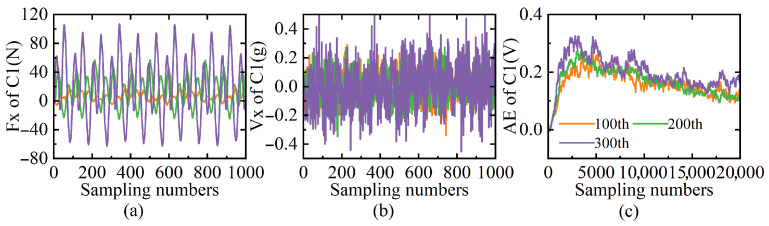
Curves of C1 in (**a**) cutting force signal; (**b**) vibration signal; (**c**) AE signal.

To explore the correlations between sensor signals and tool wear, this study first validates the effectiveness of time-domain features. Taking tool C1 as an example, Figure 3 visualizes the temporal characteristics of cutting force, vibration, and AE signals at three critical wear stages (100th, 200th, and 300th cutting cycles). It should be noted that in Figure 3c, the AE signal exhibits a different sampling number compared to the cutting force and vibration signals. This difference arises because the AE signal demonstrates minimal local variations within short sampling intervals. However, as the number of sampling points increases, distinct changes emerge in the AE signal characteristics, reflecting its unique property of being primarily sensitive to transient events rather than continuous processes. The results show that the waveform amplitudes progressively increase with the number of cutting cycles, leading to a direct rise in both peak and RMS values. These time-domain variations exhibit state-sensitive discriminative patterns. This phenomenon may correlate with enhanced cutting resistance caused by material deformation. Consequently, multi-dimensional feature extraction is employed to enhance the distinguishability of patterns between different wear stages, facilitating robust tool wear prediction modeling.

**Figure 4:**
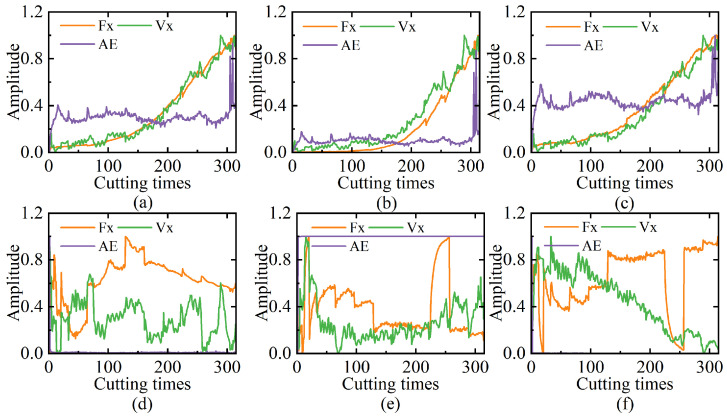
Curves of C1 in features from different domains: (**a**) standard deviation; (**b**) variance; (**c**) peak-to-peak; (**d**) frequency variance; (**e**) energy ratio 1; (**f**) energy ratio 2.

Building upon the established time-domain correlations, this study evaluates the feasibility of multi-sensor feature extraction using the multi-domain methodology in Table 1, which systematically extracts 9 time-domain features, 3 frequency-domain components, and 16 wavelet packet energy ratios. A representative subset of C1 tool features is selected for validation, with normalized distributions visualized in Figure 4. It should be noted that the aforementioned characteristics of the AE signal lead to the distinctive patterns observed in Figure 4d,e: the frequency-domain and partial time–frequency features extracted from the AE signal predominantly maintain zero values or remain constant. The analysis reveals two critical observations: (1) all features exhibit progressive variations aligned with tool wear progression, confirming their sensitivity to machining state changes; (2) individual feature trajectories show insufficient wear state specificity due to overlapping value ranges between adjacent stages. To address this limitation, the MSMDT prediction model implements coordinated parallel processing of heterogeneous features, enabling simultaneous analysis of time-domain, frequency-domain, and time–frequency-domain characteristics. This integrated approach demonstrates the capability to establish robust correlations between multi-sensor features and tool wear evolution.

### 4.3. Ablation Experiment

The prediction accuracy of the proposed method for tool wear prediction demonstrates significant dependence on multi-domain feature selection and model architecture parameters. To quantify these influences, ablation studies systematically evaluate four critical variables: (1) sensor type integration, (2) feature-domain combinations, (3) position embedding effectiveness, and (4) number of attention heads.

#### 4.3.1. Ablation Experiment on Multi-Sensor Signal Input

**Figure 5:**
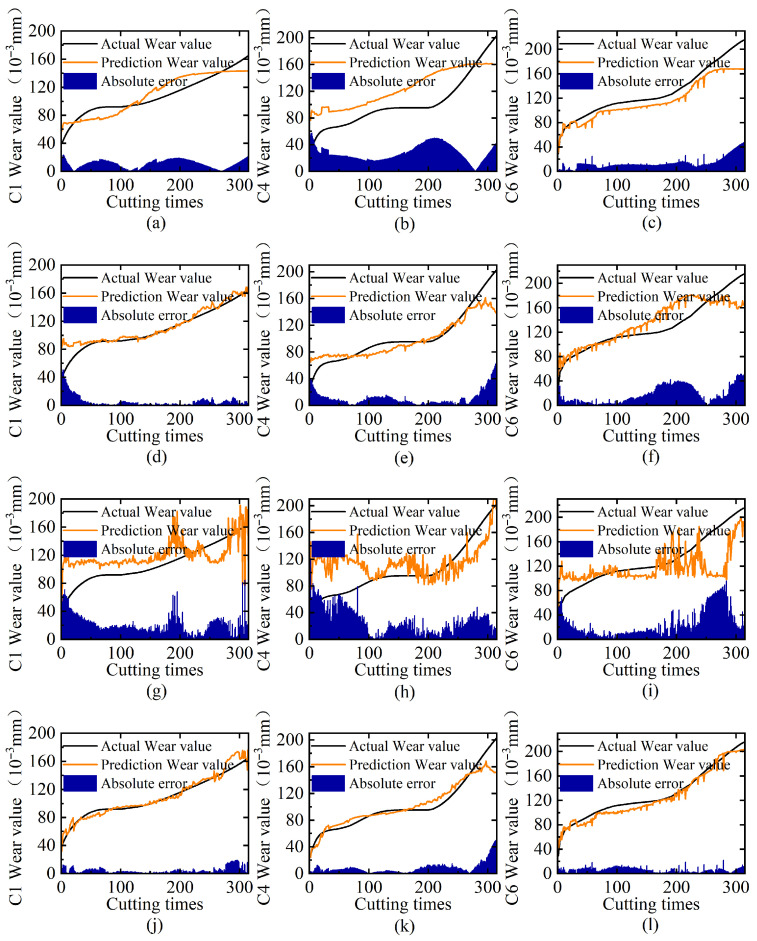
Ablation analysis of multi-sensor input: (**a**–**c**) prediction results on C1, C4, and C6 tool test sets using only cutting force sensor; (**d**–**f**) corresponding results using only vibration sensor; (**g**–**i**) corresponding results using only AE sensor; (**j**–**l**) corresponding results using multi-sensor fusion.

To systematically validate the superiority of multi-sensor fusion in tool wear monitoring, we conduct rigorous comparative experiments across three tool cases (C1/C4/C6) with four sensing configurations: individual cutting force, vibration, and AE sensors versus their fused combination. Table 3 shows the results of different sensor types, and Figure 5 displays the comparison of predicted tool wear versus the ground truth, including trend curves and prediction errors.

The experimental results reveal significant performance differences among different single-sensor signal inputs. It can be seen that the vibration sensor demonstrates superior performance to the cutting force sensor, while the AE sensor yields the least favorable results. This phenomenon is also clearly reflected by the area size enclosed by the error curve shown in Figure 5. The vibration sensor achieves its optimal performance on C1 with an MAE of 10.81. By employing multi-sensor signals, this value decreases to 4.47, representing a performance improvement exceeding 50%. The results demonstrate that the multi-sensor input strategy consistently outperforms single-sensor approaches, effectively compensating for individual sensor limitations through complementary data integration, with substantial enhancements in both MAE and RMSE compared to single-sensor methods.

#### 4.3.2. Ablation Experiment on Multi-Domain Feature Selection

**Figure 6:**
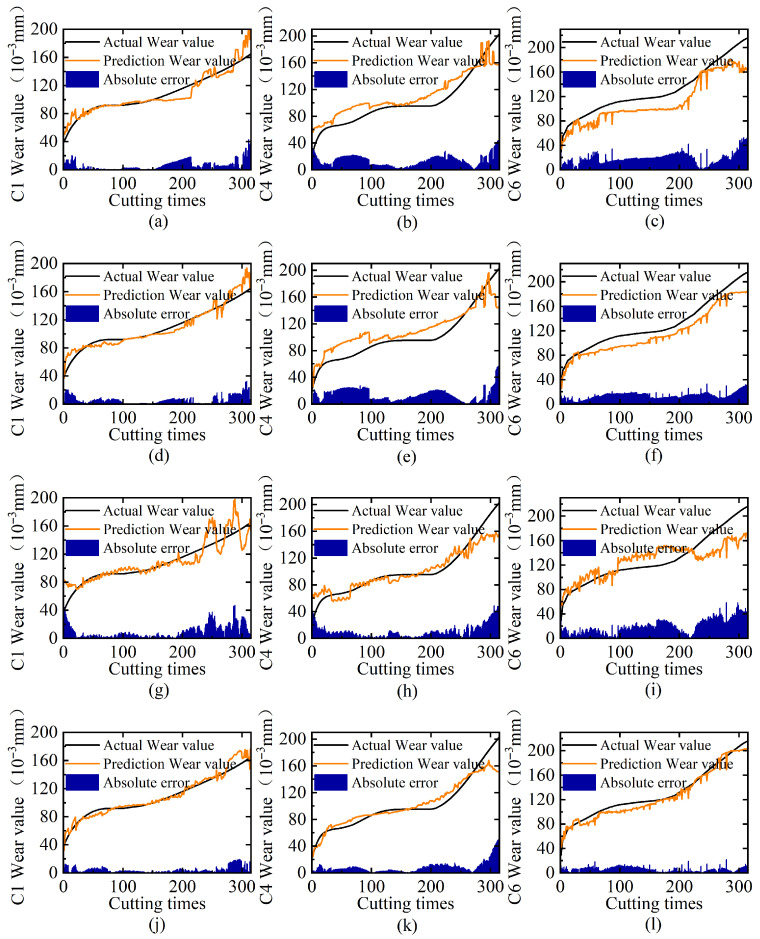
Ablation analysis of multi-domain features: (**a**–**c**) prediction results on C1, C4, and C6 tool test sets using only time-domain features; (**d**–**f**) corresponding results using only frequency-domain features; (**g**–**i**) corresponding results using only wavelet time–frequency-domain features; (**j**–**l**) corresponding results using multi-domain feature fusion.

To rigorously assess the complementary benefits of multi-domain feature fusion, we conduct controlled experiments across three tool wear cases (C1/C4/C6) under four configurations: individual time-domain, frequency-domain, and wavelet time–frequency-domain feature inputs versus their combined multi-domain fusion. All experiments maintain identical network architectures and hyperparameters, with performance quantified through MAE and RMSE metrics. Table 4 shows the results of different feature domains, and Figure 6 displays the comparison of predicted tool wear versus the ground truth, including trend curves and prediction errors.

The experimental results demonstrate distinct performance characteristics among features from different domains. The single time-domain and frequency-domain features achieve optimal performance for tool C1 but exhibit significant degradation for tools C4 and C6, indicating their limited capability in capturing complex wear patterns. In contrast, the wavelet time–frequency features demonstrate superior stability across different tools, maintaining MAE values around 10, which suggests their sensitivity to signal noise and transient events. However, the proposed multi-domain approach in this study yields the highest prediction accuracy, achieving an average MAE reduction of 3.74 across all three tools, with the lowest MAE of 4.47 observed for tool C1. Furthermore, the visualization results in Figure 6 demonstrate that the tool wear curve predicted using the multi-domain feature fusion strategy exhibits consistent variation trends with the actual wear while showing the smallest area enclosed by the error curve. These findings confirm that multi-domain feature fusion effectively integrates complementary advantages: time-domain trends capture gradual wear progression, frequency components identify periodic variations, and wavelet coefficients localize abrupt transitions.

#### 4.3.3. Ablation Experiment on Position Embedding

**Figure 7:**
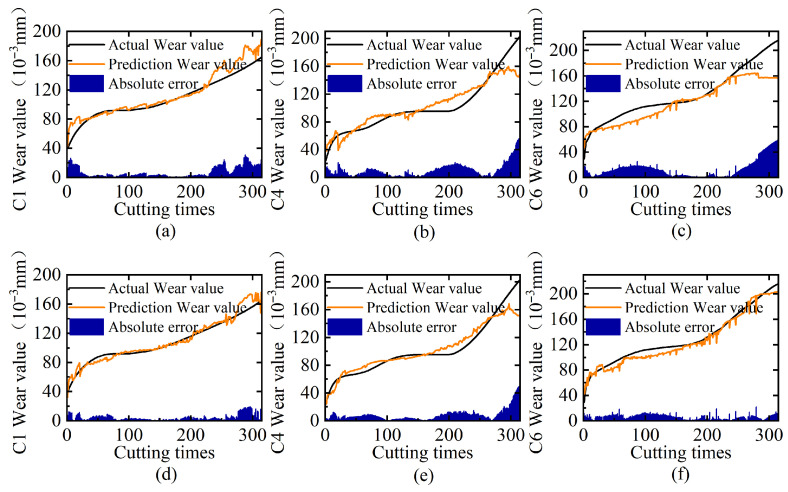
Ablation analysis of position embedding: (**a**–**c**) prediction results on C1, C4, and C6 tool test sets with standard position embedding; (**d**–**f**) corresponding results without position embedding.

In natural language processing (NLP), Transformers require position embeddings to encode word order dependencies. However, for tool wear monitoring, multi-sensor systems capture temporally synchronized signals from different sources without inherent sequential relationships. In other words, tool wear progression represents a continuous physical process monitored through simultaneously sampled sensor signals. Therefore, we eliminate the use of position embedding in this study. To validate our proposed position embedding elimination strategy (Section 3.3.1), we conduct rigorous comparative experiments across three tool cases (C1/C4/C6) using two configurations: one with position embeddings and one without. Table 5 shows the ablation results of position embedding, and Figure 7 displays the comparison of predicted tool wear versus the ground truth, including trend curves and prediction errors.

The experimental results demonstrate the significant advantages of eliminating position embedding for tool wear state prediction. Comparative analysis reveals that the prediction model without position embedding outperforms the fixed position embedding model, with MAE reductions of 39.15% for C1 and 19.02% for C4 and even a remarkable 54.54% improvement for the most challenging case C6. The visualization results in Figure 7 further confirm high consistency between the wear values predicted by the position-embedding-free MSMDT and the ground truth measurements. These findings indicate that our framework maintains good performance across different sensor configurations through its self-attention mechanism. The results validate that removing position embedding prevents artificial spatial bias injection, enables pure correlation learning through attention weights, and ensures architectural extensibility for practical deployment.

#### 4.3.4. Ablation Experiment on Multi-Head Attention

The number of heads in multi-head attention significantly impacts model performance, as it determines the capacity for learning diverse feature representations. To systematically investigate this relationship, we evaluate prediction performance across three distinct tool cases (C1/C4/C6) using varying numbers of attention heads (1, 4, 7, 14, and 28). Quantitative comparisons are presented in Table 6.

The experimental results demonstrate that the number of attention heads in the multi-head attention mechanism differentially affects prediction accuracy across different tool cases. As shown in Table 6, the best performance metrics for each tool are highlighted in bold. Specifically, the model demonstrates varying optimal configurations depending on both the tool case and evaluation metric: it achieves its lowest MAE of 4.47 on tool C1 with 14 attention heads while attaining minimum MAE values of 7.90 on C4 and 6.05 on C6 using 28 heads. However, for RMSE evaluation, the model shows superior performance on both C4 and C6 with the 14-head configuration. These variations reflect inherent engineering variability in industrial settings (e.g., tool installation tolerances) rather than methodological limitations. Based on this comprehensive analysis of prediction accuracy across all metrics and tool cases, the 14-head configuration is ultimately adopted due to its global advantages: achieving the optimal MAE on C1 (4.47) and optimal RMSE on both C4 (12.06) and C6 (7.19) while balancing computational efficiency and stability.

In summary, after sufficient ablation experiments, it is shown that multi-sensor signal inputs combined with a unified multi-domain feature extraction approach can effectively utilize the complementary information between sensors, and the use of the Transformer structure without positional embedding enables the model to autonomously learn the correlation features between different signals and tool wear state, thus achieving better prediction results.

### 4.4. Comparison with Other Methods

To comprehensively evaluate the effectiveness of the proposed MSMDT framework, we conduct systematic comparisons with various baseline methods across three distinct tool cases (C1/C4/C6). The compared methods include the following: (1) traditional machine learning methods such as Linear Regression (LR) and Support Vector Regression (SVR); (2) deep learning architectures such as Multi-layer Perceptron (MLP) [40], CNN, RNN, LSTM, and Deep LSTMs; (3) specialized hybrid LSTM-based methods for tool wear monitoring including CNN-LSTM [41], CABLSTM [42], BiLSTM [32], and CNN-BiLSTM [20]; and (4) the recent Sparse Stacked Autoencoder (SSAE) approach [43]. Table 7 presents detailed MAE and RMSE metrics for all compared methods under identical experimental conditions.

The experimental results demonstrate that the proposed MSMDT framework demonstrates superior overall performance compared to conventional machine learning and deep learning approaches. In addition, the performance of MSMDT is outstanding compared to most LSTM variants. While CNN-BiLSTM shows slightly better results for the C4 tool case, our method achieves the lowest MAE and RMSE values for both the C1 (MAE: 4.47, RMSE: 6.35) and C6 (MAE: 6.06, RMSE: 7.19) tool cases. This represents significant improvements (19.17% and 8.34% for C1 and 30.02% and 39.27% for C6, respectively) over CNN-BiLSTM. This improvement validates the Transformer architecture’s enhanced capability in capturing long-range wear progression patterns compared to recurrent networks. The experimental results collectively demonstrate that the proposed MSMDT framework achieves optimally balanced performance across all evaluation metrics, significantly outperforming conventional approaches. This superior performance stems from its effective utilization of complementary multi-sensor multi-domain features coupled with an inherently adaptable network architecture that ensures robust generalizability and operational extensibility under diverse machining conditions.

## 5. Conclusions

This study proposes the MSMDT model based on the Transformer architecture for tool wear prediction. This method extracts multi-domain features, including time-domain, frequency-domain, and wavelet time–frequency-domain features, from multi-sensor signals and performs deep fusion. It fully exploits the complementarity of multi-sensor heterogeneous data and effectively captures key information highly relevant to tool wear states. The MSMDT adopts a feature fusion encoder without position embedding, which can efficiently process and integrate multi-sensor multi-domain features in parallel, thereby improving the accuracy and robustness of tool wear prediction. Experimental results demonstrate that the proposed method outperforms other state-of-the-art methods, offering a robust solution for tool wear monitoring.

The integration of a unified multi-domain feature extraction strategy for multi-sensor heterogeneous signals with a position-embedding-free Transformer architecture demonstrates exceptional adaptability to integrated multi-sensor monitoring systems in practical industrial scenarios, while providing significant advantages across multiple critical dimensions for deployment. By systematically integrating complementary time-domain, frequency-domain, and time–frequency-domain representations, the MSMDT framework adapts to diverse sensor configurations without requiring architectural modifications, making it suitable for real-world manufacturing environments where sensor setups may vary. Feature fusion based on physically interpretable characteristics facilitates deep learning-driven predictions and enhances the understanding of tool wear mechanisms. Additionally, dimension-optimized feature representations and parallelizable Transformer operations ensure computational efficiency, achieving superior performance while maintaining high model extensibility. These attributes make MSMDT suitable for future real-time tool wear monitoring systems.

While the current study validates the MSMDT framework on a publicly available dataset, future research will focus on deploying and testing the proposed method on real CNC machining platforms to evaluate its performance under dynamic industrial conditions. This implementation study will specifically examine the framework’s robustness against real-world variabilities in tooling, workpiece materials, and machining parameters. Moreover, we will further explore additional discriminative features and advanced network architectures to enhance prediction accuracy while maintaining real-time processing capabilities. Efforts will also be devoted to optimizing computational efficiency for edge device implementation, enabling seamless integration with smart manufacturing systems. These developments aim to bridge the gap between laboratory validation and industrial application, ultimately improving the practicality and reliability of tool wear monitoring in production environments.

## Figures and Tables

**Table 2:** Experimental groups.

Group	Train Set	Test Set
1	C4, C6	C1
2	C1, C6	C4
3	C1, C4	C6

**Table 3:** Performance comparison of different sensor approaches.

Sensor Type	C1		C4		C6	
	**MAE**	**RMSE**	**MAE**	**RMSE**	**MAE**	**RMSE**
Cutting force sensor	17.43	22.62	27.74	30.27	13.80	16.82
Vibration sensor	10.81	12.30	10.75	15.79	12.36	15.04
AE sensor	20.77	25.26	26.90	34.14	24.77	33.52
Multi-sensor	**4.47**	**6.35**	**8.27**	**12.06**	**6.06**	**7.19**

*Note:* Bold values indicate better performance.

**Table 4:** Performance comparison of different feature domains.

Feature Domain	C1		C4		C6	
	**MAE**	**RMSE**	**MAE**	**RMSE**	**MAE**	**RMSE**
Time domain	6.30	9.34	13.75	16.24	18.88	21.37
Frequency domain	5.60	8.13	14.34	17.45	14.29	15.38
Time–frequency domain	9.45	13.60	10.29	14.40	10.87	15.26
Multi-domain	**4.47**	**6.35**	**8.27**	**12.06**	**6.06**	**7.19**

*Note:* Bold values indicate better performance.

**Table 5:** Performance comparison with and without position embedding.

	C1		C4		C6	
	**MAE**	**RMSE**	**MAE**	**RMSE**	**MAE**	**RMSE**
With position embedding	7.39	10.21	10.46	14.61	13.33	19.46
Without position embedding	**4.47**	**6.35**	**8.47**	**12.06**	**6.06**	**7.19**

*Note:* Bold values indicate better performance.

**Table 6:** Performance comparison of different multi-head attention configurations.

Heads	C1		C4		C6	
	**MAE**	**RMSE**	**MAE**	**RMSE**	**MAE**	**RMSE**
1	5.17	6.49	8.19	12.34	14.80	17.92
4	7.20	9.26	11.50	13.19	7.81	9.43
7	4.73	**5.97**	11.70	14.22	13.13	15.61
14	**4.47**	6.35	8.27	**12.06**	6.06	**7.19**
28	5.82	9.37	**7.90**	12.11	**6.05**	8.44

*Note:* Bold values indicate better performance.

**Table 7:** Performance comparison with other methods.

Method	C1		C4		C6	
	**MAE**	**RMSE**	**MAE**	**RMSE**	**MAE**	**RMSE**
LR	24.4	31.1	16.3	19.3	24.4	30.9
SVR	15.6	18.5	17.0	19.6	24.9	31.5
MLP	24.5	31.2	18.0	20.0	24.8	31.4
CNN	9.31	12.19	11.29	14.59	34.69	40.48
RNN	13.1	15.6	16.7	19.7	25.5	32.9
LSTM	19.6	23.9	15.6	20.8	25.3	32.4
Deep LSTMs [40]	8.3	12.1	8.7	10.2	15.2	18.9
CNN-LSTM [41]	11.18	13.77	9.39	11.85	11.34	14.33
CABLSTM [42]	7.47	8.17	-	-	-	-
BiLSTM [32]	12.8	14.6	10.9	14.2	14.7	17.7
CNN-BiLSTM [20]	5.53	6.93	**7.70**	**10.10**	8.66	11.84
SSAE [43]	-	6.66	-	11.59	-	8.49
**MSMDT (Ours)**	**4.47**	**6.35**	8.27	12.06	**6.06**	**7.19**

*Note:* Bold values indicate better performance. ‘-’ indicates that the result is not reported in the study.

## Data Availability

Data were derived from public domain resources. The data presented in this study are available in the 2010 PHM Society Conference Data Challenge at https://ieee-dataport.org/documents/2010-phm-society-conference-data-challenge (accessed on 5 June 2025), reference number [39]. These data were derived from the following resources available in the public domain: https://ieee-dataport.org/documents/2010-phm-society-conference-data-challenge (accessed on 5 June 2025).
